# Diazepam and ethanol differently modulate neuronal activity in organotypic cortical cultures

**DOI:** 10.1186/s12868-019-0540-6

**Published:** 2019-12-10

**Authors:** Matthias Kreuzer, Paul S. García, Verena Brucklacher-Waldert, Rebecca Claassen, Gerhard Schneider, Bernd Antkowiak, Berthold Drexler

**Affiliations:** 1 Department of Anesthesiology and Intensive Care, Klinikum rechts der Isar, Technical University of Munich, School of Medicine, Munich, Germany; 2 Department of Anesthesiology, Neuroanesthesia Division, Columbia University Medical Center, New York Presbyterian Hospital, New York, USA; 3 Dept. of Anesthesiology and Intensive Care, Experimental Anesthesiology Section, University Hospital Tübingen, Tübingen, Germany; 4 Werner Reichardt Center for Integrative Neuroscience, Tübingen, Germany; 5 Present Address: Horizon Discovery, 8100 Cambridge Research Park, Waterbeach, Cambridge, CB25 9TL UK; 6 Present Address: Psychiatrie-Zentrum Linthgebiet, Standort Rapperswil, Untere Bahnhofstrasse 11, 8640 Rapperswil, Switzerland

**Keywords:** Diazepam, Ethanol, GABA_A_ receptors, Benzodiazepines, Neocortex

## Abstract

**Background:**

The pharmacodynamic results of diazepam and ethanol administration are similar, in that each can mediate amnestic and sedative-hypnotic effects. Although each of these molecules effectively reduce the activity of central neurons, diazepam does so through modulation of a more specific set of receptor targets (GABA_A_ receptors containing a γ-subunit), while alcohol is less selective in its receptor bioactivity. Our investigation focuses on divergent actions of diazepam and ethanol on the firing patterns of cultured cortical neurons.

**Method:**

We used electrophysiological recordings from organotypic slice cultures derived from Sprague–Dawley rat neocortex. We exposed these cultures to either diazepam (15 and 30 µM, n = 7) or ethanol (30 and 60 mM, n = 11) and recorded the electrical activity at baseline and experimental conditions. For analysis, we extracted the episodes of spontaneous activity, i.e., cortical up-states. After separation of action potential and local field potential (LFP) activity, we looked at differences in the number of action potentials, in the spectral power of the LFP, as well as in the coupling between action potential and LFP phase.

**Results:**

While both substances seem to decrease neocortical action potential firing in a not significantly different (p = 0.659, Mann–Whitney U) fashion, diazepam increases the spectral power of the up-state without significantly impacting the spectral composition, whereas ethanol does not significantly change the spectral power but the oscillatory architecture of the up-state as revealed by the Friedman test with Bonferroni correction (p < 0.05). Further, the action potential to LFP-phase coupling reveals a synchronizing effect of diazepam for a wide frequency range and a narrow-band de-synchronizing effect for ethanol (p < 0.05, Kolmogorov–Smirnov test).

**Conclusion:**

Diazepam and ethanol, induce specific patterns of network depressant actions. Diazepam induces cortical network inhibition and increased synchronicity via gamma subunit containing GABA_A_ receptors. Ethanol also induces cortical network inhibition, but without an increase in synchronicity via a wider span of molecular targets.

## Background

Diazepam and ethanol are widely used central depressants with similar pharmacological properties. Behaviorally, they produce sedation, amnesia and, at higher concentrations, unconsciousness. These effects are at least partially mediated by neurons in the cerebral cortex. Both agents significantly reduce the excitability of cortical neurons when administered within a behaviorally relevant range of concentrations [1, 2]. Besides their common properties, distinct differences do exist. Diazepam almost exclusively binds to GABA_A_ receptors containing α1-, α2-, α3-, or α5-subunits typically together with a γ-subunit [3]. Different behavioral effects of benzodiazepines can be attributed to specific GABA_A_ receptor subtypes, e.g., sedation by diazepam is mediated via GABA_A_ receptors containing the α1-subunit [4, 5]. Ethanol on the other hand is less selective in its molecular targets. In addition to GABA_A_ receptors containing δ-subunits, glutamate receptors, GABA_B_ receptors, and potassium channels present other pre- and postsynaptic targets. Ethanol affects receptor trafficking through changes e.g. in NMDA receptor phosphorylation and also neurosteroid synthesis is influenced by alcohol [6–14].

Thus, based on the differential molecular targets of diazepam and ethanol, it seems reasonable to assume that their effects on network activity are also discriminable. Indeed, electroencephalographic features of event related potentials differ between ethanol and diazepam [15], but a detailed examination of the drug-induced differences in the activity of neocortical neuronal populations is still missing. Neocortical networks, consisting of pyramidal cells and inhibitory interneurons are capable of generating oscillations in the theta and gamma frequency range either due to external input or due to their intrinsic network properties [16, 17]. It is possible that enhancing the strength of inhibitory synapses by diazepam not only results in a decrease of average discharge rates but also modifies correlated firing of cortical neurons.

Synchronous oscillatory activity in the neocortex is a form of correlated neuronal firing that is involved in working memory tasks and sensorimotor integration [18]. Electroencephalogram recordings in vivo can help to investigate neuronal synchrony in neocortical oscillatory activity. But the impact of subcortical structures like the thalamus on these oscillations is difficult to interpret. Ex vivo models lacking subcortical structures, e.g., cultured brain slices from the neocortex [19], present an approach to evaluate the spontaneous neuronal activity recorded as local field potential (LFP) in the isolated neocortex. The neocortex is among the most important structures in the brain to induce sedation and general anesthesia by benzodiazepines and ethanol [20]. For that reason, we decided to probe for differential actions of diazepam and ethanol in organotypic neocortical slice cultures from rats. The firing patterns of cultured cortical neurons are characterized by phases of high-frequency action potential firing, called up-states, halted by neuronal silence, termed down states [21–23]. In order to identify substance-specific effects on spontaneous up-state activity, we evaluated the change of power spectral density (PSD) of the up-states as well as the changes in synchronization between action potentials (AP) and the phase of the LFP-up-state using the analytical signal.

## Methods

### Preparation of organotypic cortical slice cultures

All procedures were approved by the Animal Care Committee (Eberhard-Karls-University, Tuebingen, Germany) and were in accordance with the institutional and federal guidelines of the German Animal Welfare Act (TierSchG). We put in a great deal of effort to reduce the number and suffering of animals. We prepared organotypic slice cultures from the neocortex of P3–5 rats as described earlier [19, 24].

In brief, six P3–P5 Sprague–Dawley rat pups of both sexes (Charles River, Sulzfeld, Germany) were put into a see-through plastic container and anesthetized with 4 vol% halothane using high air flow (Draeger Vapor 19.3, Draegerwerk, Luebeck, Germany). Animals were decapitated well after loss of righting reflex, but before cardio-respiratory depression occured. We withdrew the cortical hemisphere, removed the meninges, and cut 300 µm thick coronal slices, which we transferred onto glass coverslips and embedded them in a plasma clot. We transferred the coverslips into plastic tubes containing 750 µL of nutrition medium (consisting of horse serum, Hank’s balanced salt solution, basal medium Eagle, glutamine and glucose) to be incubated in a roller drum at 37 °C. After 1 day in culture, we added antimitotics (pyrimidine analog and DNA synthesis inhibitor) and we renewed the suspension and the antimitotics twice a week. For our experiments, we used the cultures after 2 weeks in vitro.

### Electrophysiologic recordings

We performed the extracellular multi-unit recordings in a recording chamber mounted on an inverted microscope. Therefore, we perfused the slices with artificial cerebrospinal fluid (aCSF) consisting of (in mM) NaCl 120, KCl 3.3, NaH_2_PO_4_ 1.13, NaHCO_3_ 26, CaCl_2_ 1.8 and glucose 11, bubbled with 95% oxygen and 5% carbon dioxide. We positioned aCSF-filled glass electrodes with a resistance of about 3 to 5 MΩ on the surface of the slices and advanced into the tissue until extracellular spikes exceeding 100 µV in amplitude were visible. All experiments were conducted at 34 °C. For preparation of the test solutions we dissolved diazepam (B. Braun, Melsungen, Germany) and ethanol (99%, university pharmacy) in the aCSF to yield the desired concentration. We applied the drugs (diazepam or ethanol) via bath perfusion using syringe pumps (ZAK, Marktheidenfeld, Germany) at a flow rate of approximately 1 mL min^−1^. After switching to experimental drug-containing solutions, at least 95% of the medium in the experimental chamber was replaced within 2 min. Effects on the spike patterns were stable about 5 min later. To ensure steady state conditions, we carried out the recordings 10 min after commencing the change of the drug-containing perfusate using a personal computer with the Digidata 1200 AD/DA interface and Axoscope 9 software (Axon Instruments, Union City, CA).

### Separation of local field potential and action potential activity and signal preprocessing

**Fig. 1:**
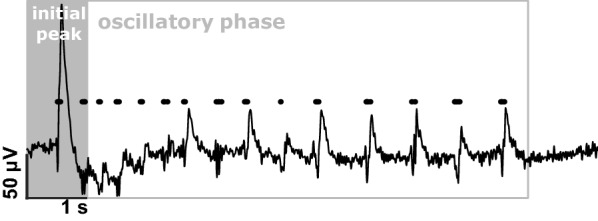
Exemplary trace of a recorded cortical up-state. The early phase is dominated by a strong initial peak followed by a slow transient phase back to the baseline amplitude. Following this initial peak oscillatory activity develops. For our analyses, we excluded the initial peak segment and focused on the oscillatory phase. The black dots indicate the occurrence of action potentials

We included n = 7 and n = 11 cultures in the diazepam and ethanol group, respectively. For each culture, we recorded spontaneous LFP activity during control conditions as well as in the presence of either ethanol or diazepam. The recorded electrophysiological data was band-pass filtered to separate AP activity from LFP activity. Filter settings for AP traces were 200–2000 Hz. For the identification of AP spikes and their time of occurrence we used a self-programmed MATLAB routine. The routine annotates the time point of a spike based on a set amplitude threshold that was defined as three times the standard deviation of baseline noise. We also used MATLAB to extract episodes of cortical up-state activity from the LFP recordings. Prior to extraction of the up-states, we resampled the LFP to 500 Hz. We only used recordings with valid data for all concentration levels to have a paired design for statistical analysis. Figure 1 presents a representative LFP with corresponding spiking activity.

### Action potential frequency

We plotted the cumulative probability of the frequencies of action potential firing in the first 200 ms of the cortical up-state for each condition. The analysis was based on the action potentials detected by the threshold-based routine. Therefore, we used the empirical cumulative distribution function plot (*cdfplot*) function in MATLAB.

### Analysis of local field potential activity

The recorded LFP present the cumulative activity of neuronal activity in proximity of the recording electrode. We restricted our analyses to cortical up-states longer than 2.5 s to be able to adequately characterize the spectral composition of the oscillatory phase after the initial peak. We excluded the first second of the up-state, i.e., the initial peak from the spectral analysis because of its very dominant amplitude and its non-oscillatory behaviour. Hence, we evaluated the features of the initial peak separately. Figure 1 describes our approach. We measured the peak-to-peak amplitude of the initial up-state to quantify possible drug-induced effects. For the analysis of the initial up-state amplitude, we had to exclude one diazepam experiment because we only observed short up-states in one concentration stage of this recording. For the same reasons, we excluded four ethanol experiments.

Further, we excluded the last 0.2 s of each up-state to prevent a bias due to the transition back to a cortical down-state at the end of the up-state.

We used the MATLAB *pmtm* function that applies the Thomson’s multitaper method with 256 data points and time-halfbandwidth product to default for PSD calculation. We also calculated the normalized PSD (nPSD), by dividing the total power by the sum of power between 2 and 30 Hz. While this approach provides information regarding changes in the spectral distribution with increasing drug concentrations, we used the information of AP times and LFP phase to evaluate possible changes in AP to LFP-phase locking.

### Action potential probability at distinct field potential phase

We assessed the LFP phase with the Hilbert transform [25]. Using this method, an analytical signal *X*(*t*) is generated from the original trace, here the LFP up-state episode. *X*(*t*) is complex and the real part complies with the original trace and the imaginary part is the original trace after a ninety-degree phase shift. The analytic signal corresponds to the envelope of the original trace. The analytic phase *Φ*(*t*) can be obtained from $\phi (t) = \arctan \frac{{x_{IM} (t)}}{{x_{RE} (t)}}.$ In order to correctly determine $\phi (t)$, the trace has to be filtered to a narrow frequency range. Here, we analysed frequencies up to 16 Hz in non-overlapping 2 Hz steps. We followed a 5-degree raster of binning the AP to the phase. By matching the AP to the analytic phase we are able to evaluate possible (de-) synchronizing effects between AP and LFP-phase.

### Statistical analysis

To describe diazepam- or ethanol-induced effects on cortical up-state activity we applied different statistical approaches. To statistically describe possible changes in peak-to-peak amplitude of the initial LFP-spike, the number of AP, as well as in PSD and nPSD, we applied the Friedman test with pairwise Wilcoxon signed rank tests and a Bonferroni correction. For unpaired comparisons, we used the Mann–Whitney U test. For outlier analysis, we applied the MATLAB *isoutlier* function, defining elements that are greater than three scaled median absolute deviations away from the median as outlier. For changes in PSD and nPSD we only considered changes to be significant if they occurred in at least two neighboring frequencies [26]. We used Kolmogorov–Smirnov test to find differences in the probability distribution of AP frequency. Being aware of the limited sample size in our experiments, we supplemented the signed rank test with Hedges’ g tests as effect size using the MATLAB-based MES toolbox [27]. We further used the Kolmogorov–Smirnov test to detect changes in the distribution of action potentials in relation to the LFP phase as well as differences in the distribution of AP frequency.

We performed all descriptive and inference statistical tests with MATLAB. We used the MATLAB *boxplot* function for visualization of the data. In the boxplots the central horizontal line indicates the median whereas lower and upper box limits indicate the 25th and 75th percentiles. The whiskers span between the most extreme data points not considered outliers.

## Results

### Effects of diazepam and ethanol on action potential firing of cultured cortical neurons

The depression of neocortical spike activity by diazepam and ethanol had been reported earlier [1, 2]. In the current study, the number of spikes significantly decreased for both diazepam (Χ^2^ = 8; p = 0.0183; n = 7) and ethanol (Χ^2^ = 11.17; p = 0.0013 n = 11), excluding one outlier in the ethanol group (Additional file 1: Figure S1A). For the diazepam experiments, the spike rate per 180 s recording time was 2070 [1470 4654] (median and 1st and 3rd quartile) for control conditions, 1259 [950 1541] for 15 µM, and 740 [579 904] for 30 µM diazepam. For the ethanol experiments, the spike rate was 2009 [824 2798] at control conditions, 1076 [435 1703] in the presence of 30 mM ethanol, and 673 [253 2210] with 60 mM ethanol. Table 1 contains the detailed statistical information regarding the substance-induced effects. In short, diazepam significantly and/or strongly reduced the firing rate in a concentration-dependent fashion. Ethanol significantly reduced the AP rate, but did not have this concentration-dependent effect.

**Table 1:** p-Values and effect sizes for the comparisons between the concentration levels of diazepam and ethanol for the depression of action potentials (AP), the number of LFP up-states, and the up-state duration

	CNT vs. 15 μM		CNT vs. 30 μM		15 vs. 30 μM	
	p	g	p	G	p	g
Diazepam						
AP depression	0.078	0.89 [0.34 1.94]; strong	0.031	1.20 [0.70 2.59]; strong	0.0156	1.20 [0.72 2.57]; strong
# of up-states	0.063	1.18 [0.53 2.70]; strong	0.031	1.33 [0.78 2.84]; strong	0.141	0.20 [0.02 0.84]; weak
Up-state duration	0.031	− 0.88 [− 0.43 − 1.77]; strong	0.11	− 1.25 [− 0.54 − 2.75]; strong	0.578	− 0.04 [− 0.80 0.47]

	CNT vs. 30 mM		CNT vs. 60 mM		30 vs. 60 mM	
	p	g	p	G	p	g
Ethanol						
AP depression	0.054	0.43 [− 0.08 1.16]	0.003	0.69 [0.31 1.26]; medium	0.042	0.22 [− 0.11 0.51]
# of up-states	0.079	0.75 [0.13 1.60]; medium	0.311	0.43 [− 027 1.28]	1	− 0.28 [− 0.70 0.12]
Up-state duration	0.432	− 0.14 [− 0.82 0.32]	0.0488	0.40 [− 0.11 1.05]; weak	0.0195	0.56 [0.28 1.05]; medium

**Fig. 2:**
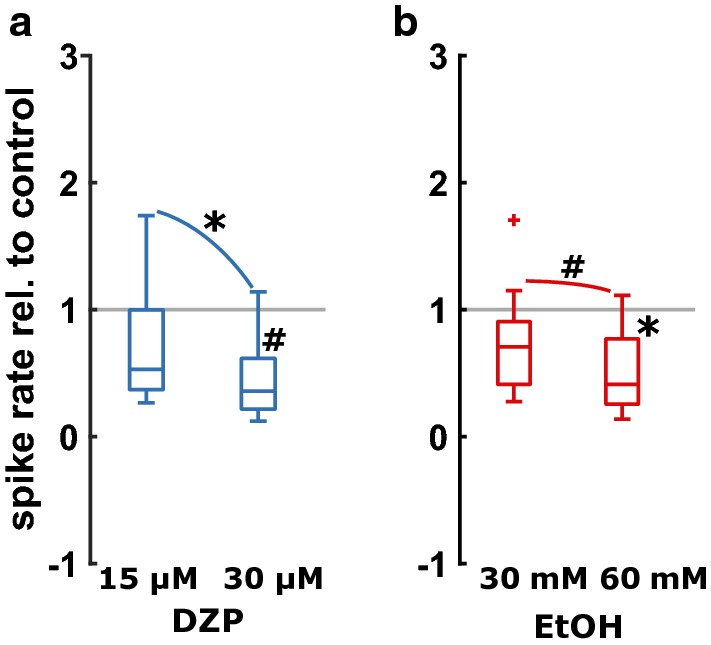
Action potentials for **a** diazepam (DZP) and **b** ethanol (EtOH) relative to control conditions. Both diazepam (blue, left) and ethanol (red, right) decreased the number of action potentials in a concentration-dependent manner. **a** 30 µM diazepam had a strong effect on the spiking rate compared versus control conditions. Diazepam caused a decrease in the number of action potentials as indicated by Hedge’s g (g = 1.20 [0.70 2.59]) that was not significant after Bonferroni correction (p = 0.031, uncorrected). The decrease in spike rate from 15 µM to 30 µM diazepam was significant and strong (p = 0.0156; g = 1.20 [0.72 2.57]). **b** When compared to control conditions, 30 mM ethanol did not show a significant reduction of spike rate (p = 0.054, g = 0.43 [− 0.08 1.16]), but 60 mM significantly reduced the spiking rate (p = 0.003; g = 0.69 [0.31 1.26]). The change from 30 mM to 60 mM ethanol was weak and not significant after Bonferroni correction (p = 0.042, uncorrected; g = 0.22 [− 0.11 0.51]). *p < 0.05 Bonferroni corrected; ^#^p < 0.05 uncorrected

The boxplots in Fig. 2 depict the relative change in the number of spikes which was 53% [37% 100%] for 15 µM and 36% [22% 62%] for 30 µM diazepam when compared to control conditions. The relative reduction of spike rate when compared to control conditions was 71% [41% 91%] for 30 mM and 41% [26% 77%] for 60 mM ethanol. Even though we could observe a substance-induced reduction of AP, there was no significant difference in the reduction of spiking activity for the low concentrations of diazepam (15 µM) and ethanol (30 mM) versus the respective control conditions (p = 0.659, Mann–Whitney U). Hence, we considered these concentrations as nearly equipotent.

**Fig. 3:**
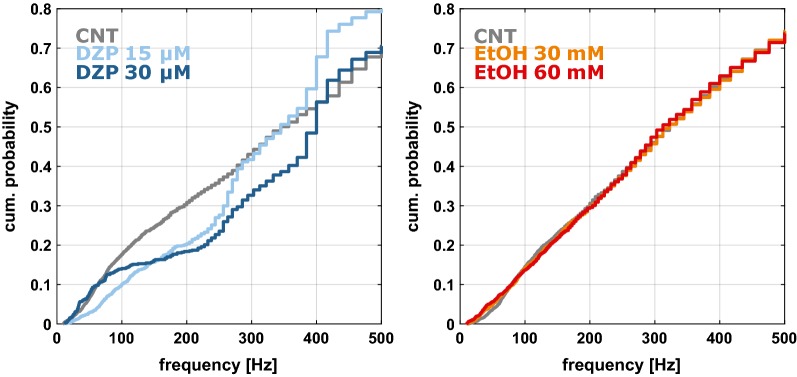
Cumulative probability plots for the action potential frequency distribution in the first 200 ms of each up-state for diazepam (left) and ethanol (right). Diazepam significantly affected this distribution, whereas ethanol did not. p < 0.001 for all comparisons between the diazepam groups (control vs. 15 µM; control vs. 30 µM; 15 µM vs. 30 µM). For the ethanol experiments the test results were p = 0.799 (cnt vs. 30 mM); p = 0.364 (cnt vs. 60 mM); and p = 0.867 (30 mM vs. 60 mM)

Diazepam, in contrast to ethanol caused a significant change in the spiking frequency throughout the initial 200 ms of the up-state. For all comparisons in the diazepam group (cnt vs. 15 µM; cnt vs. 30 µM; 15 µM vs. 30 µM) we found a p < 0.001; For the ethanol experiments the test results were p = 0.799 (cnt vs. 30 mM); p = 0.364 (cnt vs. 60 mM); and p = 0.867 (30 mM vs. 60 mM). Figure 3 displays the corresponding cumulative probability plots.

### Actions of diazepam and ethanol on the number of neuronal up-states

**Fig. 4:**
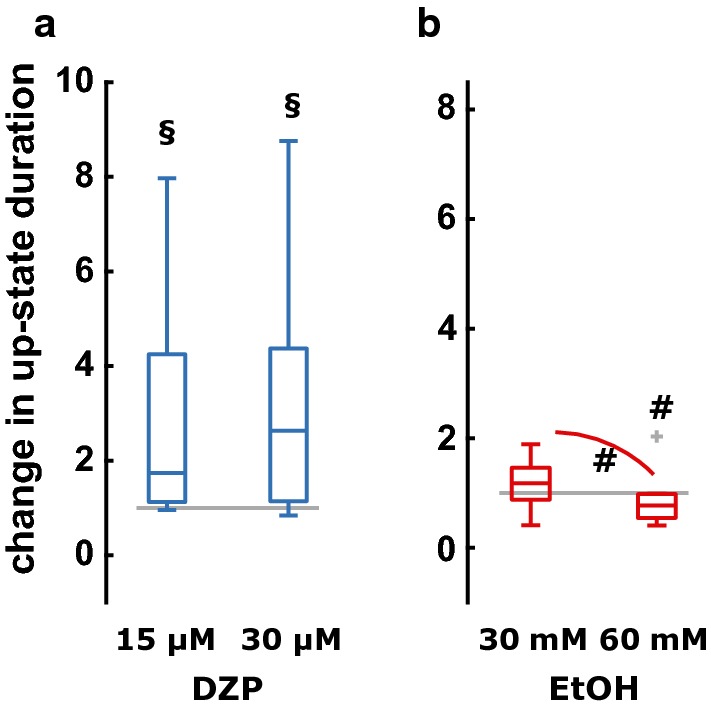
Relative change in the duration of up-states for **a** diazepam (DZP) and **b** ethanol (EtOH). **a** Diazepam had a strong effect on the duration of up-states when compared versus control conditions (15 µM: (p = 0.031, uncorrected; g = − 0.88 [− 0.43 − 1.77]); 30 µM diazepam (p = 0.11; g = − 1.25 [− 0.54 − 2.75], and p = 0.578 and g = − 0.04 [− 0.80 0.47] for 15 µM vs. 30 µM diazepam. **b** While 30 mM ethanol had no effect on up-state duration when compared versus control conditions (p = 0.432; g = − 0.14 [− 0.82 0.32]), 60 mM ethanol had a weak, but significant (p = 0.0488, uncorrected) effect (g = 0.40 [− 0.11 1.05]) on up-state duration when compared versus control conditions. Further, 60 mM ethanol had a medium effect causing shorter up-states (p = 0.0195, uncorrected; g = 0.55 [0.28 1.05), when compared against 30 mM ethanol. ^#^p < 0.05 uncorrected; ^§^strong effect

Diazepam significantly reduced the number of up-states from 28 [12 44] during control to 5 [4.3 19] at 15 µM and 4 [3.3 12.8] at 30 µM (p = 0.011; Χ^2^ = 8.96). At the same time, the up-state duration did not reveal a significant difference among groups (p = 0.1561, Χ^2^ = 3.71), but the effect size analysis revealed a strong effect of 15 µM and 30 µM diazepam on up-state duration as presented in Table 1. The median up-state duration was 2.3 s [1.5 3.4] s at control conditions, 4.3 s [2.6 8.0] s at 15 µM, and 6.0 [3.0 10.2] at 30 µM diazepam. Figure 4 displays the relative change in up-state duration by diazepam and ethanol, respectively. For the investigation of the effect of ethanol we did not observe a significant change in the number up-states (p = 0.174; Χ^2^ = 3.5) from 23 [14 32] at control conditions to 13.5 [8 22] at 30 mM and 14.5 [9 27] at 60 mM ethanol. The effect of ethanol on the number of up-states was medium for 30 mM ethanol and ‘fail’ for 60 mM. For the evaluation of the duration of up-states in the ethanol experiments, we had to exclude two experiments detected as outliers as shown in the boxplots in Additional file 1: Figure S1B in the supplement. Ethanol significantly affected the up-state duration (p = 0.0247, Χ^2^ = 7.4). Median up-state duration was 2.6 s [2.1 3.9] s at control conditions, 3.3 s [1.7 5.0] s with 30 mM ethanol, and 2.1 s [1.4 3.4] s with 60 mM ethanol.

### Effects of diazepam and ethanol on absolute amplitude of the initial up-state

For both substances, we did not observe a significant effect on the initial amplitude. The Friedman test revealed a p = 0.513 (Χ^2^ = 1.33) for diazepam and p = 0.687 (Χ^2^ = 0.75) for ethanol.

### Spectral properties of LFP up-states after the initial action potential

**Fig. 5:**
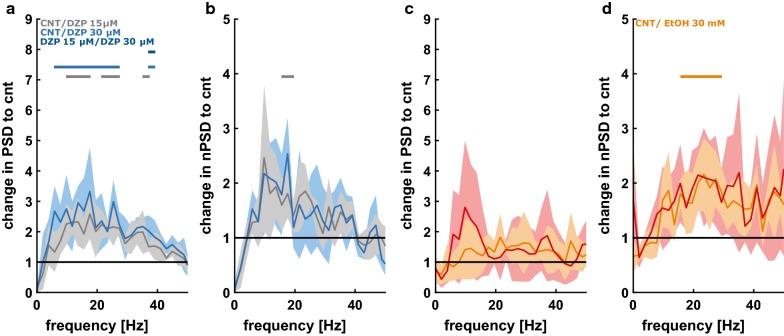
Relative changes in absolute power spectral density (PSD) or normalized PSD (nPSD) as induced by diazepam (DZP) or ethanol (EtOH). **a** DZP-induced changes in PSD: DZP concentration-dependently increases the power (i.e., the amplitude) in a wide range of frequencies. The grey and blue horizontal line indicate a significant effect of low (15 µM, grey) or high (30 µM, blue) DZP concentration vs. control (CNT). A horizontal bar in dark blue indicates a significant difference between 15 and 30 µM DZP. **b** DZP-induced changes in nPSD: The oscillatory composition did not change in a significant fashion, except for a narrow frequency range around 20 Hz for 15 µM DZP. **c** EtOH-induced changes in PSD: EtOH does not affect the power (i.e., the amplitude) in LFP oscillations. **d** EtOH-induced changes in nPSD: the oscillatory composition changed in a significant fashion towards a stronger contribution of higher frequencies above 10 Hz for the low EtOH concentration (30 mM) versus control as indicated by the horizontal bars. The solid trend lines indicate the median and the shaded areas the median absolute deviation. The horizontal bars indicate a significant difference (p < 0.05, Wilcoxon signed rank test) for the comparison indicated by the color of the bar

For diazepam we observed an increase in the PSD of the up-state episodes over the entire frequency range. We did not find a significant difference between the concentration levels. Further, the nPSD did not change significantly, indicating a preserved oscillatory architecture in the up-state. Figure 5a, b highlight these findings. We found contrasting results for ethanol, which did not significantly change the PSD of the up-states. Ethanol had an effect on nPSD in certain frequency ranges indicative of an altered oscillatory architecture of the up-states, but only for the low, 30 mM concentration. Figure 5c, d presents the findings for ethanol.

There were only changes in nPSD in the very low frequencies. For ethanol we observed PSD changes only in a limited frequency range, whereas the frequency composition as evaluated by nPSD changed as well.

### Actions of diazepam and ethanol on the AP firing to LFP phase relationship

**Fig. 6:**
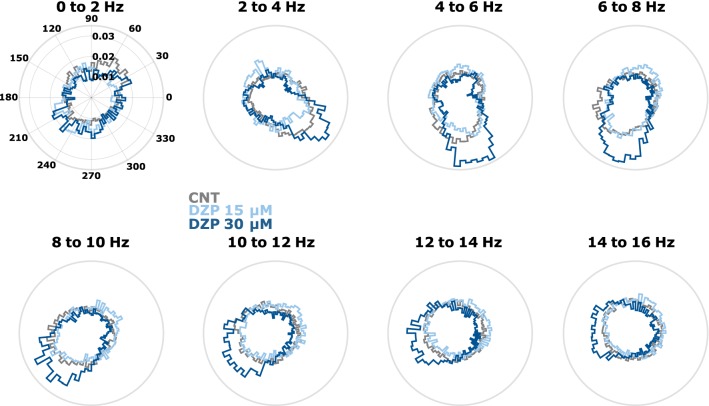
Diazepam-induced changes of the action potential to local field potential phase relationships. Especially at the high diazepam concentration (dark blue) peaks in the distribution develop that are indicative of a strong spike to phase locking. *DZP* diazepam, *CNT* control conditions

**Fig. 7:**
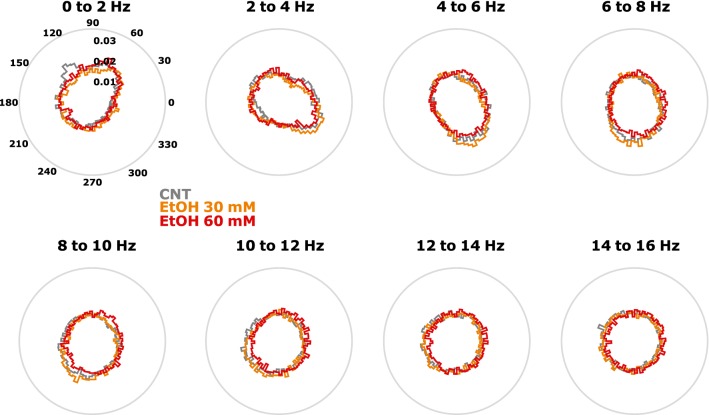
Ethanol-induced changes of the action potential to local field potential phase relationships. Application of ethanol (EtOH) leads to more uniform distribution of AP and LFP phase relationships. *CNT* control conditions

Diazepam induced a stronger effect on AP to LFP phase coupling than ethanol in the 2–16 Hz range. This effect was concentration-dependent. Higher concentrations of diazepam caused a stronger concentration of AP in a limited range of LFP phase. Ethanol in contrast did not affect the AP to LFP-relationship in this way. The effects were weaker and the high dose of ethanol caused a more uniform distribution of AP among the LFP phase. Table 2 presents the results of the statistical analysis. Figure 6 shows the polar plots of AP to LFP-phase distribution for diazepam and Fig. 7 shows the AP to LFP-phase distribution for ethanol.

**Table 2:** p-Values of the Kolmogorov–Smirnov test evaluating possible differences in the distribution of AP to LFP-phase

FREQ (Hz)	CNT vs. DZP 15 µM	CNT vs. DZP 30 µM	DZP 15 µM vs. DZP 30 µM	CNT vs. EtOH 30 mM	CNT vs. EtOH 60 mM	EtOH 30 mM vs. EtOH 60 mM
0–2	0.7409	0.2461	0.4615	*0.0105*	0.2461	0.1713
2–4	0.0484	0.0105	0.0018	0.7409	0.2461	0.4615
4–6	*0.0009*	*0.0005*	*0*	0.3427	*0.006*	*0.006*
6–8	*0.0484*	*0.0033*	*0.0001*	*0.0484*	0.3427	*0.006*
8–10	*0.018*	*0.0002*	*0*	0.1158	0.2461	*0.0033*
10–12	0.1713	*0*	*0*	0.4615	0.1158	*0.0033*
12–14	0.5982	*0*	*0.0009*	0.9561	0.1713	0.1158
14–16	0.3427	*0*	*0.0033*	0.8685	0.1158	0.1158

Diazepam (left columns) causes a concentration-dependent change towards a less uniform distribution, i.e., the AP are occurring at a higher rate at the same LFP phase. Ethanol in contrast (right columns) had a weaker, opposite effect towards a more uniform distribution of AP to LFP-phase. Significant differences are indicated as italic p-values

## Discussion

In the current study we could show that diazepam and ethanol both depress spontaneous cortical activity in cultured neocortical slices from rat. However, a detailed analysis revealed that diazepam and ethanol affect spontaneous firing patterns in a different fashion. While diazepam had a synchronizing effect on local field potential activity, ethanol only caused a small change towards desynchronization of spiking activity.

### Model system, limitations of the study, and relevance of used concentrations

We opted for organotypic cultures for the current study due to their specific characteristics: unlike acute slices where most synaptic connections are lost due to the preparation process and diffusion times of drugs can take up to hours, organotypic cultures display an intact cytoarchitecture [19], an “in vivo-like” receptor expression [28], and therefore a high level of connectivity leading to high neuronal activity. The “adult-like” developmental status of organotypic slice cultures after cultivation, including e.g. the hyperpolarizing nature of GABA was shown before [29]. Furthermore, diffusion times of drugs are short [30, 31] and allow for good environmental control. Nevertheless, organotypic cultures present a reduced model system. But based on their properties, they can serve as a bridging model between studies on expressed receptors e.g., in oocytes and in vivo recordings in animals.

Concerning possible limitations of the study there are two major questions: first, does the data from a single recording site represent the state of the rest of the network, and second, can one extrapolate the data obtained from a single site in such an extremely limited local network to an intact brain network? The goal of using OTC is to obtain findings that are applicable to an intact in vivo system. In previous studies we have shown that neuronal activity in OTC is highly synchronized, even in co-cultures from the thalamus and the cortex from rats, e.g. in Figure 3 from Ref. [32]. This issue is reviewed in detail in [33]. Therefore, it seems well appropriate to use the information from a small cortical OTC, taken as a representative snapshot of the network, to draw conclusions about network properties.

For the current ex vivo study concentrations of diazepam in the micromolar range and concentrations of ethanol in the millimolar range were chosen to induce clear cut effects. Both, diazepam in the µM range and ethanol in the mM range roughly led to a 50%-reduction of the spontaneous action potential firing rate and can therefore be considered as nearly equipotent. We previously described that a 50%-reduction of the spontaneous firing rate in rodent cultured cortical slices, induced by benzodiazepines and other common drugs of anesthesia corresponds quite well with the EC_50_ of loss of righting reflex [2], which in turn presents a surrogate measure for loss of consciousness in humans. For ethanol a spike rate 50% effective concentration of 38.6 mM in cultured murine neuronal networks has been described [34]. Furthermore, Draski et al. reported blood ethanol concentrations in a range of 64 mM to 81 mM around loss and return of the righting reflex in rats [35, 36]. A blood ethanol concentration of 0.08%, the limit of legal driving in some countries, would correspond to 17 mM [37]. Therefore, the concentrations of diazepam and ethanol used for the current study are neither “low”, nor “intoxicating” [8, 38], but correspond approximately to loss of consciousness in vivo.

### Diazepam and ethanol differently influence spiking and up-state behavior

With our analytical approach we could identify differential effects of diazepam and ethanol at concentrations inducing comparable depression of overall network activity, perhaps indicating non-overlapping molecular targets. These findings may present a consequence of diazepam and ethanol targeting different subtypes of GABA_A_ receptors [9, 10, 39]. Our analyses investigating the relationship between the instantaneous phase of the LFP and the occurrence of an action potential revealed a phase to AP synchronization with diazepam and a weaker effect towards desynchronization with ethanol. The relative change in the spectral composition of the recorded up-state activity as evaluated by our multitaper-PSD analysis supports the finding. Diazepam does not affect the architecture of up-states activity, but the amplitudes of the up-states, also an indicator of a synchronization process. Ethanol in contrast has no effect on up-state amplitude but leads to faster oscillatory activity in the up-states, as sign for desynchronization. Our results further indicated a decrease in the number of up-states with diazepam. But the fewer up-states became longer. This finding supports the results regarding network synchronization; because once an up-state was initiated the synchronized activity could maintain neuronal activity for a longer time. For ethanol, we observed a different effect. We did not observe a significant reduction in the number of up-states with ethanol, and a decrease in up-state time with 60 mM of ethanol. Ethanol-induced desynchronizing mechanisms could cause the up-state to fade earlier. The finding that ethanol shows desynchronizing properties are somehow in contrast to the work by Wilson et al. [40] in newborn mice demonstrating a hypersynchrony and an increase in LFP-oscillations by ethanol. However, in their study chronic effects of ethanol on the development of the central nervous system over a time period of several months were studied, which is in stark contrast to our study comparing the acute effects of diazepam and ethanol.

### Putative mechanisms of differential actions

Experimental studies suggest that synchronous firing of inhibitory interneuronal networks present the source of neuronal network oscillations [41–43]. Diazepam may alter the firing frequency of neurons by specifically modulating the decay of synaptic responses via specific GABA_A_ receptors (containing γ-subunits) and cause neuronal network activity to synchronize [41, 44]. This selective action of diazepam on GABA_A_ receptors may lead to an increased AP to LFP phase locking caused by (subtle) inhibitory action on neuronal network activity resulting in more synchronized firing patterns that lead to increased AP to LFP-phase locking. Interestingly, recent results from in vivo experiments in mice showed that diazepam at low, anxiolytic concentrations, and thus not causing an effect on neuronal discharge rate, leads to a decrease in theta oscillations (6–10 Hz) while cells remained significantly phase locked [45]. This observation agrees with our data showing that higher concentrations of diazepam cause a decrease in firing rate which is paralleled by an increase of AP to LFP phase locking in our ex vivo model. Other current studies described that diazepam fosters oscillations in the low gamma range (20–50 Hz) via α2-containing GABA_A_ receptors [46] and that the acetylcholine receptor agonist carbachol enhances synchronicity in cortical pyramidal cell–basket cell networks via muscarinic M1 receptors [47]. Therefore, it is tempting to speculate that the diazepam-induced increase in cortical network synchrony as observed in our study might involve α2-containing GABA_A_ receptors and muscarinic M1 receptors.

The more heterogeneous effect of ethanol may lead to effects on neuronal network activity, very different to diazepam. Further, ethanol increases GABA_A_ receptor-mediated inhibition mainly caused by δ-subunit-containing receptors, but other types may be upregulated as well [48, 49]. Increasing tonic inhibition dampened the (low-frequency) oscillatory activity of excitatory cells in an in silico model [44].

Furthermore, diazepam and ethanol may act via receptors that differ in their desensitization features. Desensitization of GABA_A_ receptors (the main molecular target of diazepam) could play a key role in altering the ability of inhibitory networks to synchronize [41]. One exception from this is the δ-subunit containing GABA_A_ receptor, which does not demonstrate desensitization. This GABA_A_ receptor subtype is diazepam-insensitive but has been proposed as a target for ethanol [10].

## Conclusion

In conclusion, we could present new evidence that the depression of spontaneous neuronal activity in the neocortex by substances inducing anxiolysis, sedation, loss of consciousness and addiction is not uniform. Depending on the specific molecular targets, diazepam and ethanol, induce specific patterns of network depressant actions. Diazepam, acting mostly through GABA_A_ receptors containing the gamma-subunit, induces cortical network inhibition and increased synchronicity, whereas ethanol, acting via a much wider range of molecular targets, also induces cortical network inhibition, but without an increase in synchronicity.

## Supplementary information

**Additional file 1: Figure S1.** We excluded one experiment (depicted by ‘+’) as outlier, as defined by the MATLAB boxplot and function, for the analyses regarding the change in the number of spikes (A) as well as two experiments regarding the duration of the up-states (B).

## Data Availability

The data used can be found in Additional file.

## Abbreviations

aCSF: artificial cerebrospinal fluid
AP: action potential
CNT: control
DZP: diazepam
EtOH: ethanol
GABA: γ-aminobutyric acid
LFP: local field potential
PSD: power spectral density
