# Supplementary figures and images for: Diazepam and ethanol differently modulate neuronal activity in organotypic cortical cultures
Source: BMC Neurosci. 2019 Dec 10;20:58. doi: 10.1186/s12868-019-0540-6 (PMC6902402; doi:10.1186/s12868-019-0540-6)

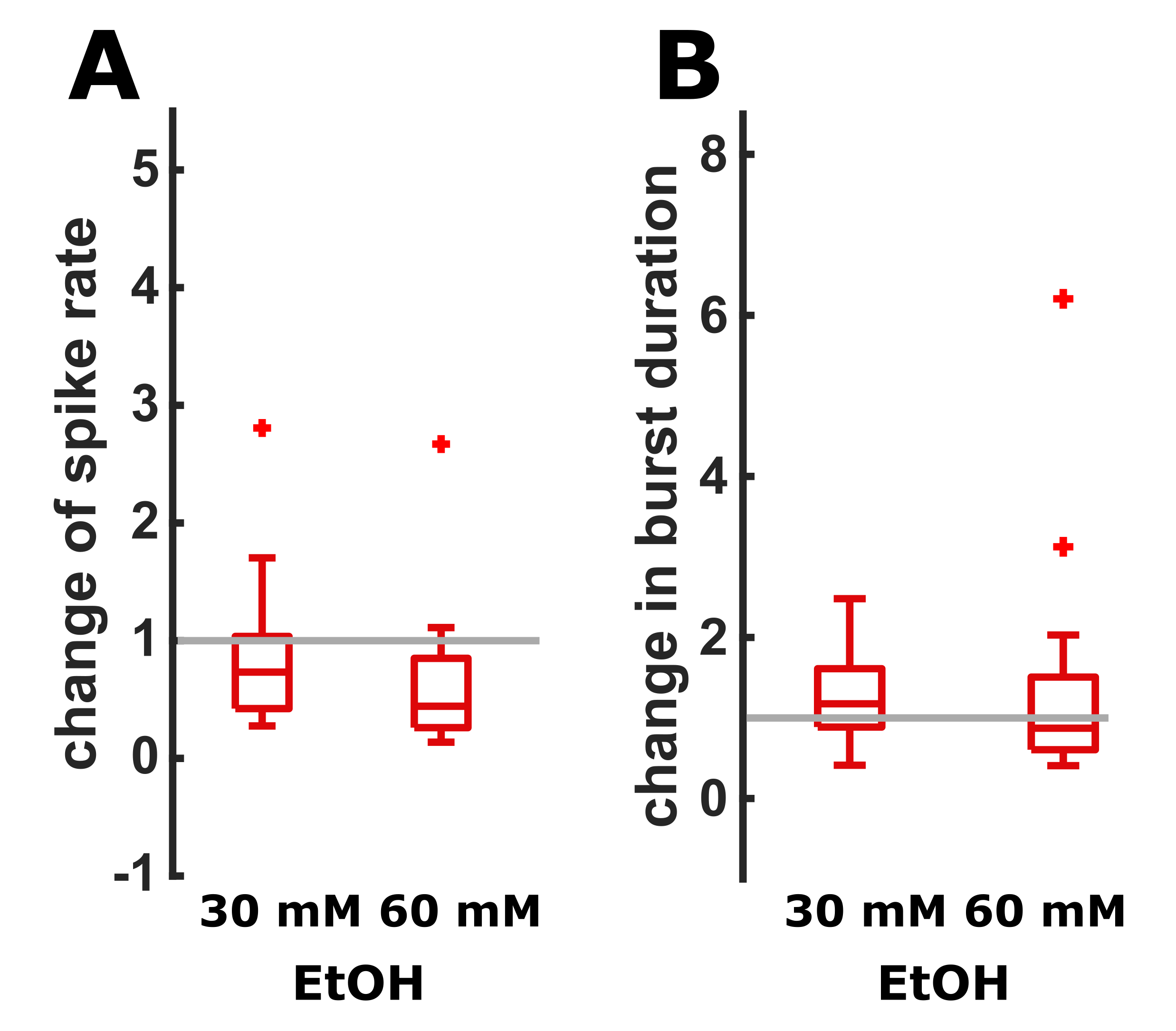

Supplement: Supplementary file 1 — Additional file 1: Figure S1. We excluded one experiment (depicted by ‘+’) as outlier, as defined by the MATLAB boxplot and function, for the analyses regarding the change in the number of spikes (A) as well as two experiments regarding the duration of the up-states (B). [file 12868_2019_540_MOESM1_ESM.png]
